# Black, Proud, Silent, and Loud: Experiences of a Junior Faculty Member in 2020

**DOI:** 10.1093/geroni/igab046.1610

**Published:** 2021-12-17

**Authors:** Candace Brown

**Affiliations:** University of North Carolina, Charlotte, Charlotte, North Carolina, United States

## Abstract

Several social injustice issues, well known within the Black community, were brought to light to other ethnic/racial groups in 2020 and could no longer be ignored within the academic community. This led to personal, departmental, and institutional initiatives meant to increase racism awareness and apply change in thought and action. These initiatives often came at a cost of personal time and resources to Black and Indigenous People of Color academics, expected to contribute to these initiatives, redefine classroom syllabi, uphold research agendas, and continue with mentoring activities amidst their home environment (due to COVID-19) while monitoring their own feelings of pride, hurt, anger, anxiousness, and often- fatigue. This presentation will present the perceived triumphs and failed experiences of a junior faculty member, how they navigated this process, and explain the continued importance of institutions’ forward movement of initiatives meant to change the social and racial academic atmosphere.

